# Supplementary material for: A Review of Bioactive Compounds and Antioxidant Activity Properties of Piper Species
Source: Molecules. 2022 Oct 10;27(19):6774. doi: 10.3390/molecules27196774 (PMC9573611; doi:10.3390/molecules27196774)
Supplement: Supplementary file 1 [file molecules-27-06774-s001.zip › molecules-1921452-supplementary.pdf]

molecules

MDPI

Supplementary Materials

2. Distribution, Botanical, Traditional Use and Pharmacological Properties of Five *Piper* Species

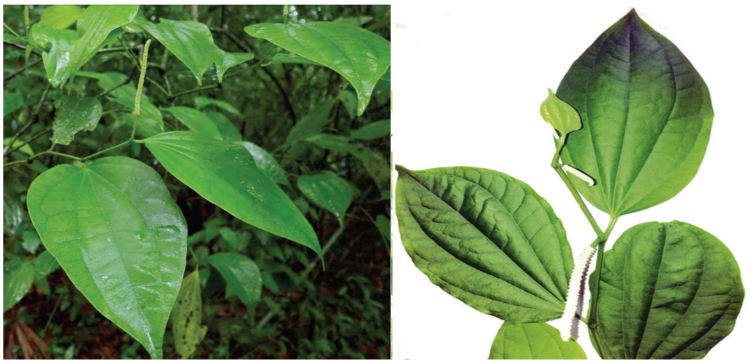

Figure S1. *P. amalago* leaves and plant

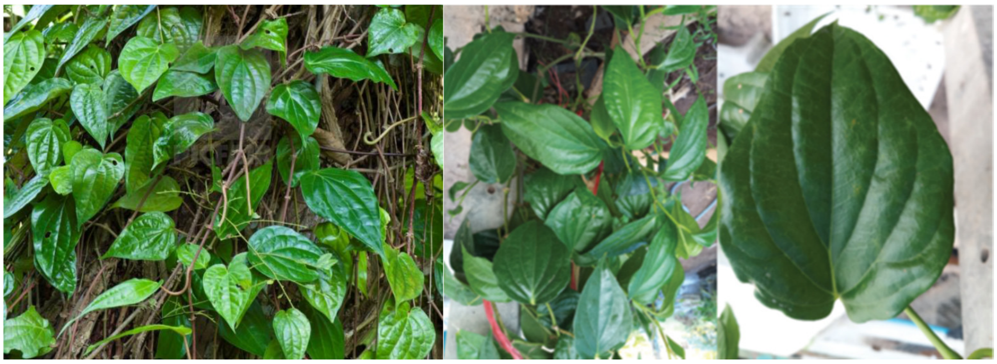

Figure S2. *P. betle* plant and leaves

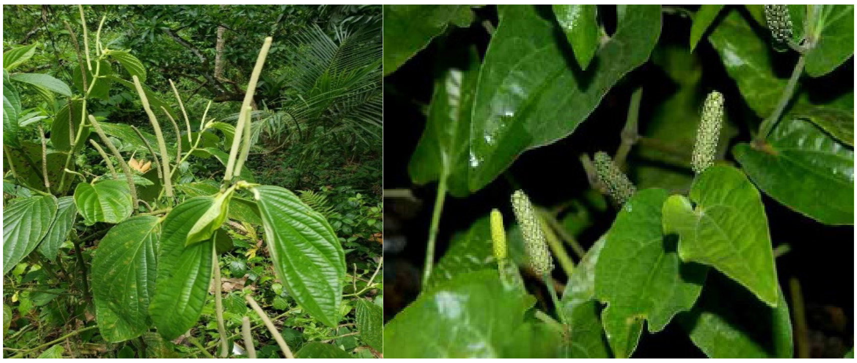

Figure S3. *P. hispidum* plant and leaves

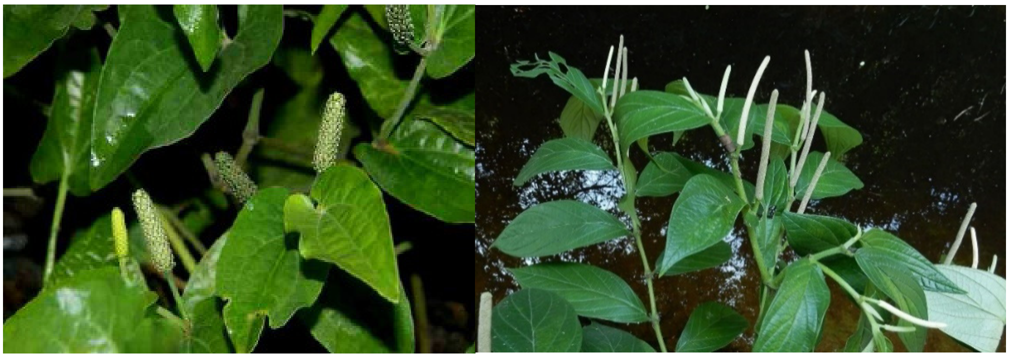

Figure S4. *P. longum* leaves and mature dried corns

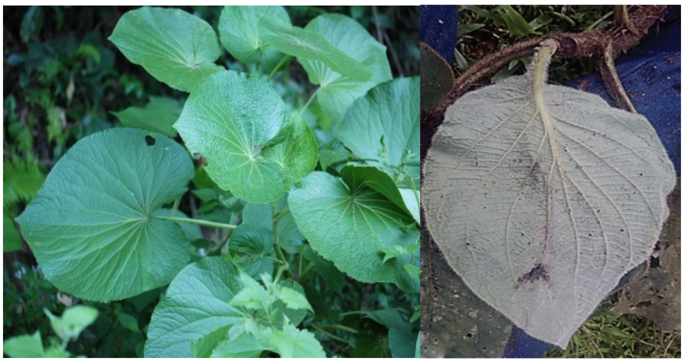

Figure S5. *P. umbellatum* plant and leaf

3. Chemical Composition of Five *Piper* Species

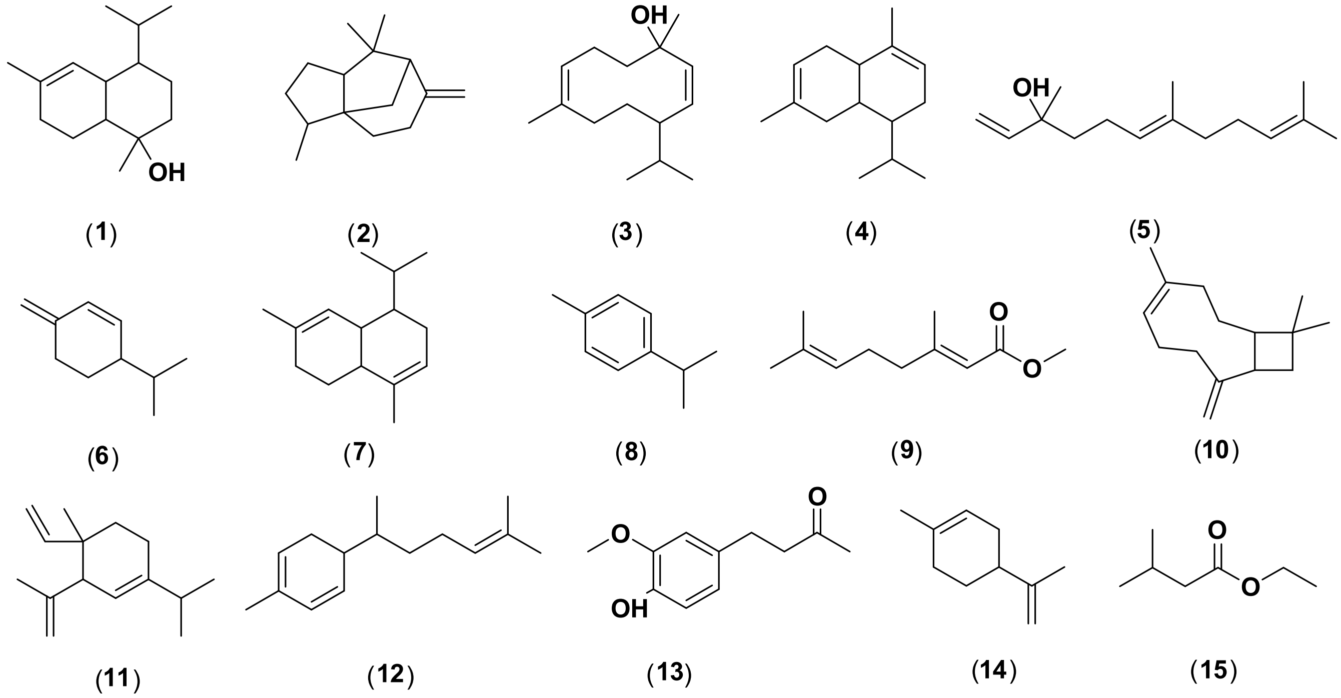

Figure S6. *Cont.*

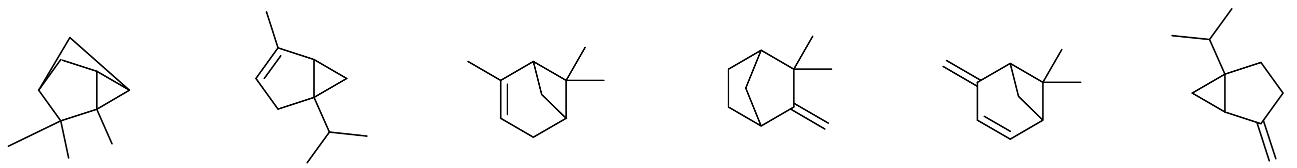

(**16**) (**17**) (**18**) (**19**) (**20**) (**21**)

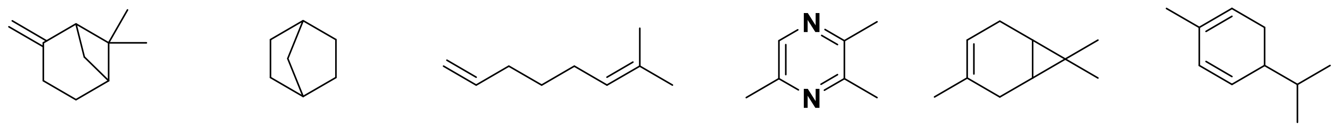

(**22**) (**23**) (**24**) (**25**) (**26**) (**27**)

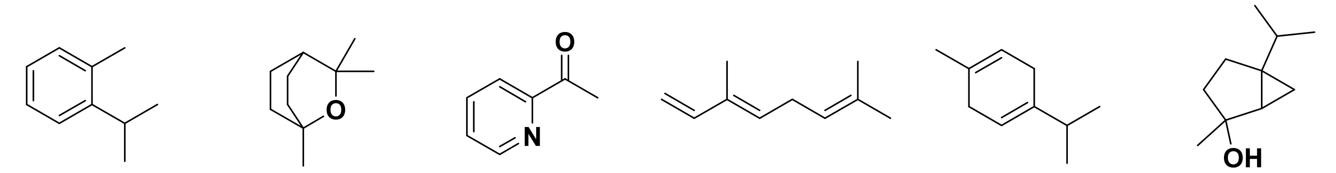

(**28**) (**29**) (**30**) (**31**) (**32**) (**33**)

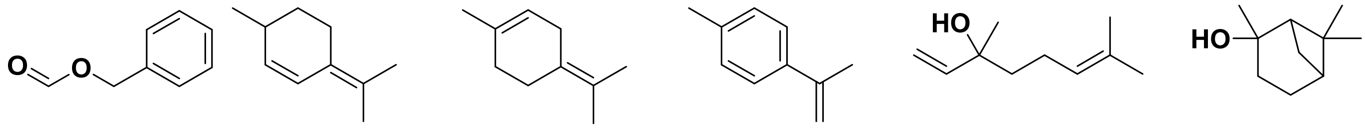

(**34**) (**35**) (**36**) (**37**) (**38**) (**39**)

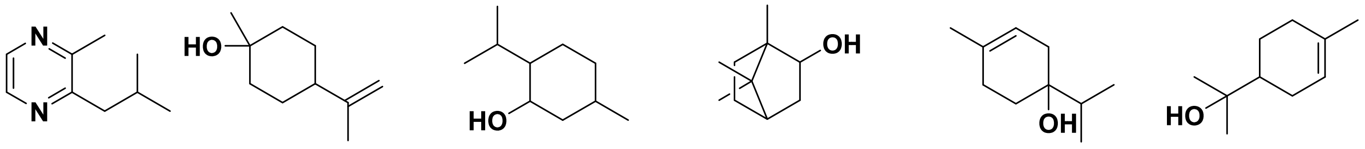

(**40**) (**41**) (**42**) (**43**) (**44**) (**45**)

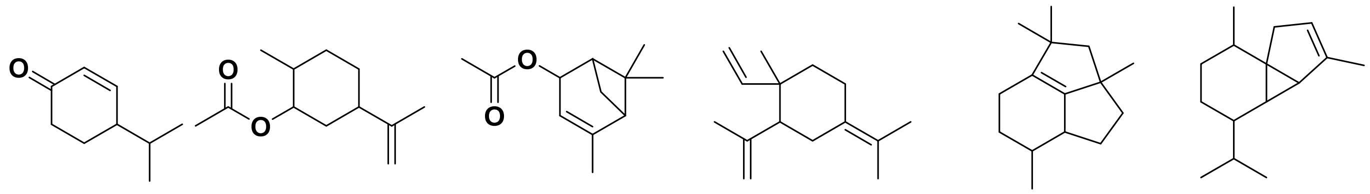

(**46**) (**47**) (**48**) (**49**) (**50**) (**51**)

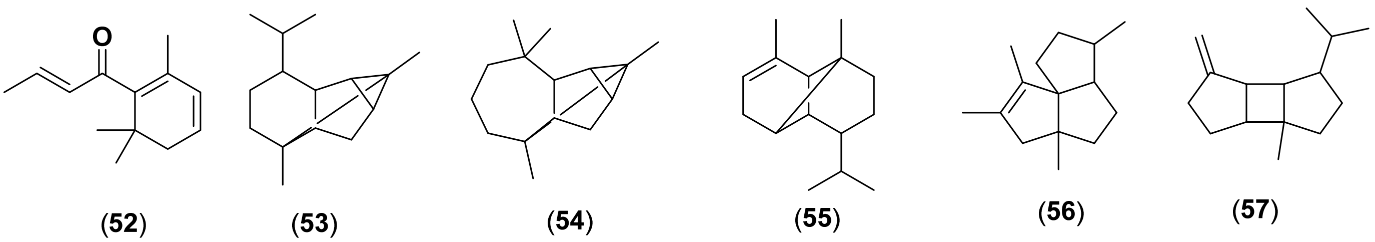

(**52**) (**53**) (**54**) (**55**) (**56**) (**57**)

Figure S6. *Cont.*

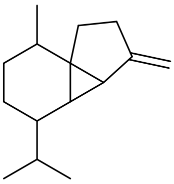
(**58**)

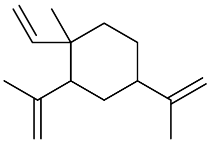
(**59**)

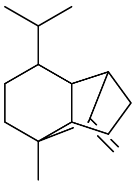
(**60**)

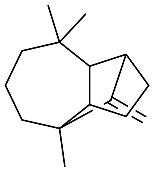
(**61**)

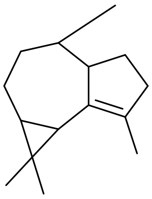
(**62**)

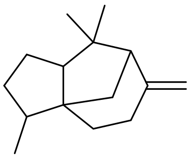
(**63**)

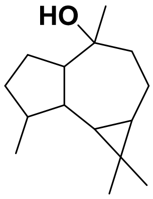

(**106**)

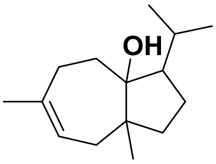

(**107**)

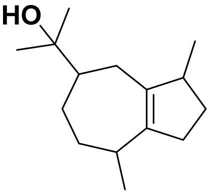

(**108**)

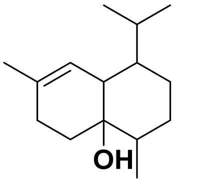

(**109**)

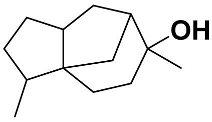

(**110**)

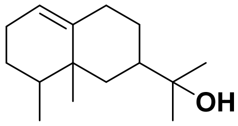

(**111**)

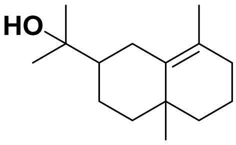

(**112**)

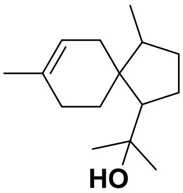

(**113**)

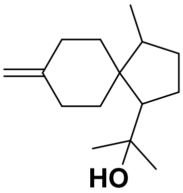

(**114**)

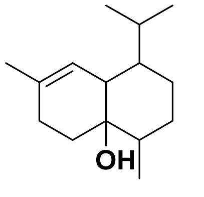

(**115**)

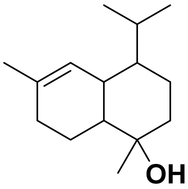

(**116**)

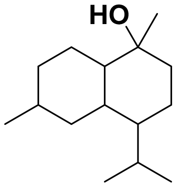

(**117**)

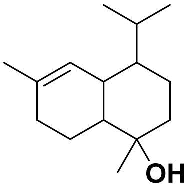

(**118**)

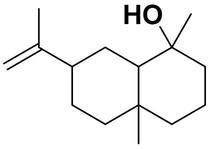

(**119**)

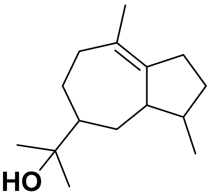

(**120**)

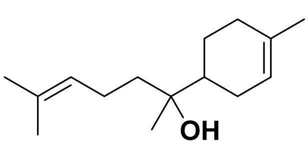

(**121**)

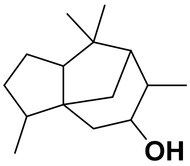

(**122**)

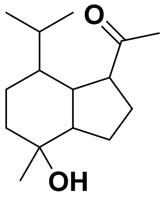

(**123**)

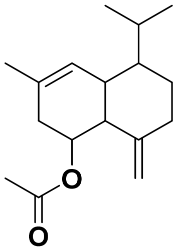

(**124**)

Figure S6. The structure chemical compound of *P. amalago*

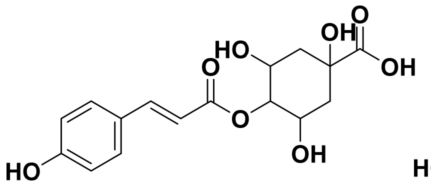
(**125**)

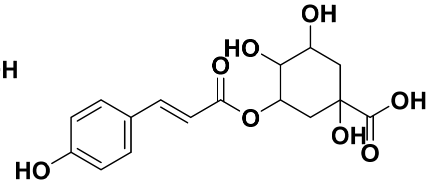
(**126**)

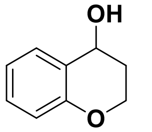
(**127**)

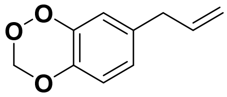
(**128**)

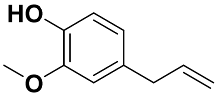
(**129**)

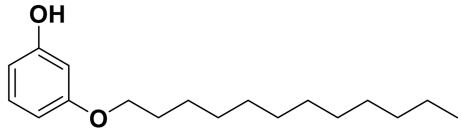
(**130**)

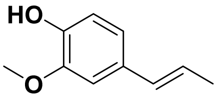
(**131**)

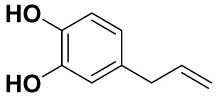
(**132**)

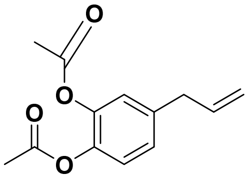
(**133**)

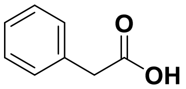
(**134**)

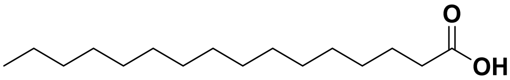
(**135**)

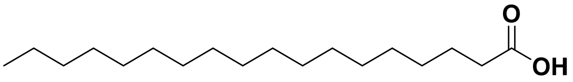
(**136**)

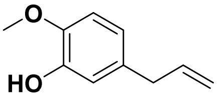
(**137**)

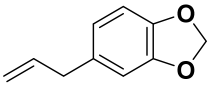
(**138**)

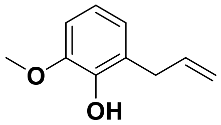
(**139**)

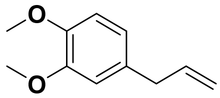
(**140**)

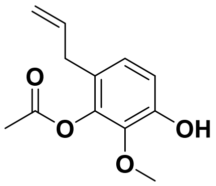
(**141**)

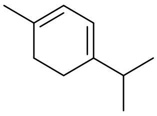
(**142**)

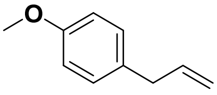
(**143**)

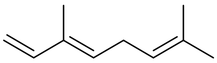
(**144**)

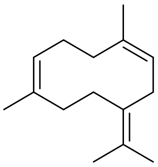
(**145**)

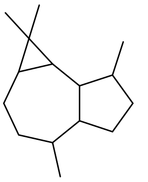
(**146**)

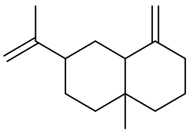
(**147**)

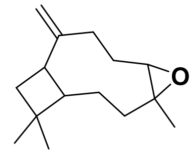
(**148**)

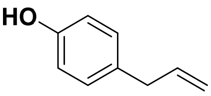
(**149**)

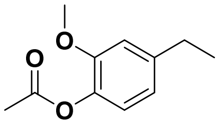
(**150**)

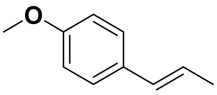
(**151**)

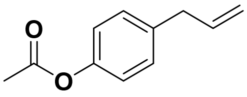
(**152**)

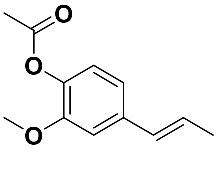
(**153**)

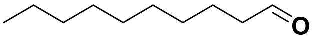
(**154**)

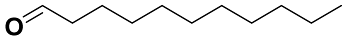
(**155**)

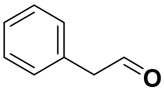
(**156**)

Figure S7. The structure chemical compound of *P. betle*

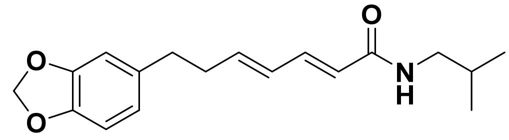

(**157**)

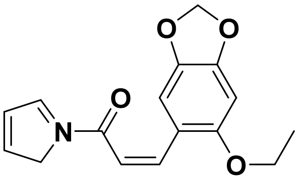

(**158**)

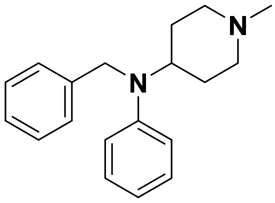

(**159**)

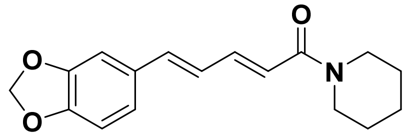

(**160**)

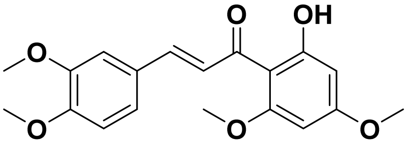

(**161**)

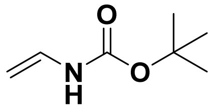

(**162**)

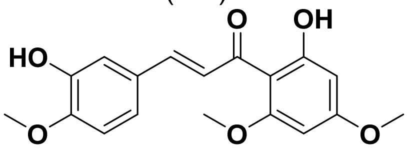

(**163**)

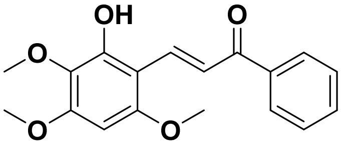

(**164**)

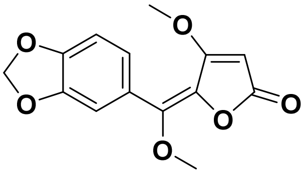

(**165**)

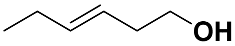

(**166**)

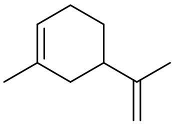

(**167**)

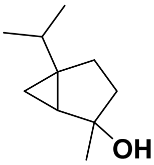

(**168**)

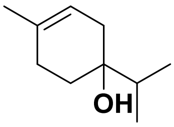

(**169**)

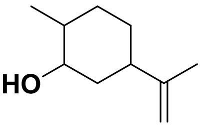

(**170**)

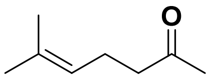

(**171**)

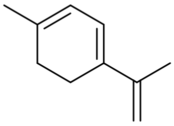

(**172**)

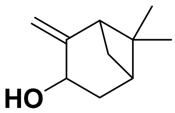

(**173**)

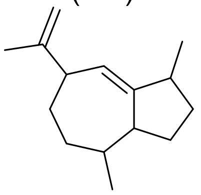

(**174**)

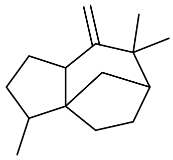

(**175**)

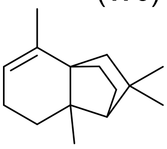

(**176**)

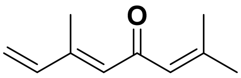

(**177**)

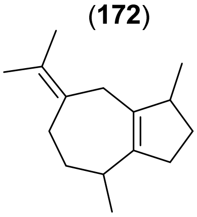

(**178**)

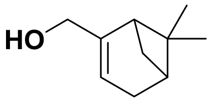

(**179**)

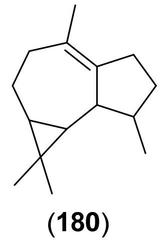

(**180**)

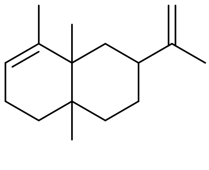

(**181**)

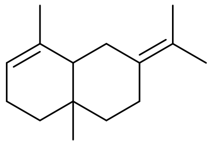

(**182**)

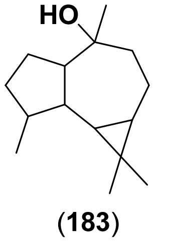

(**183**)

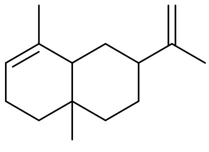

(**184**)

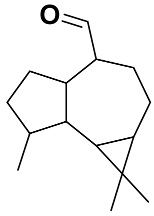

(**185**)

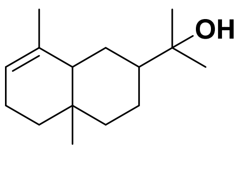

(**186**)

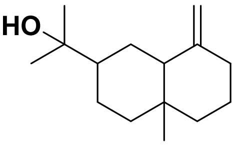

(**187**)

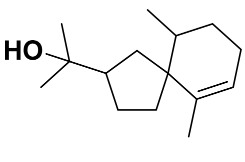

(**188**)

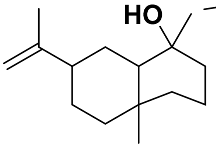

(**189**)

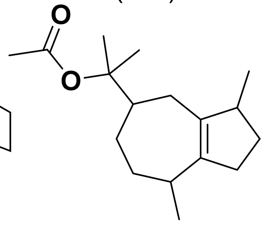

(**190**)

Figure S8. The structure chemical compound of *P. hispidum*

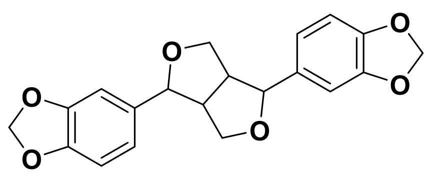

(**191**)

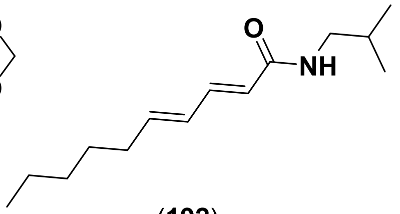

(**192**)

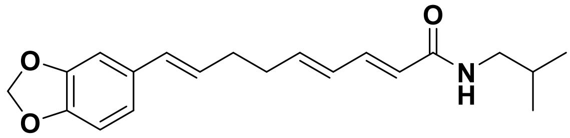

(**193**)

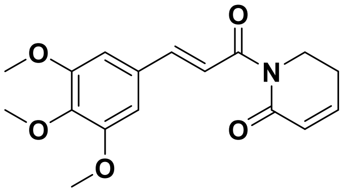

(**194**)

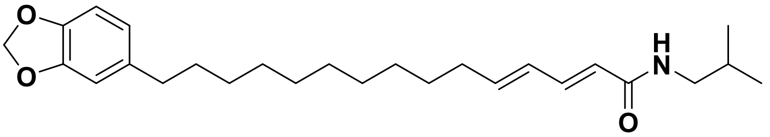

(**195**)

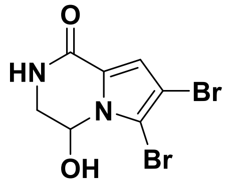

(**196**)

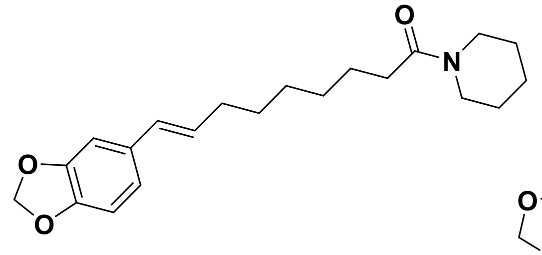

(**197**)

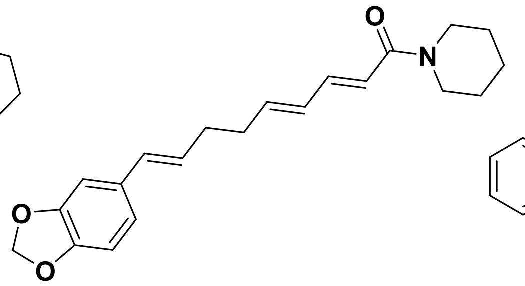

(**198**)

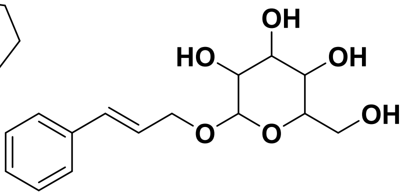

(**199**)

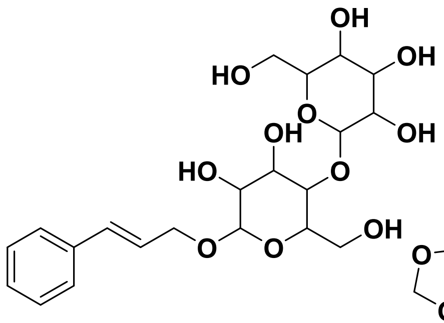

(**200**)

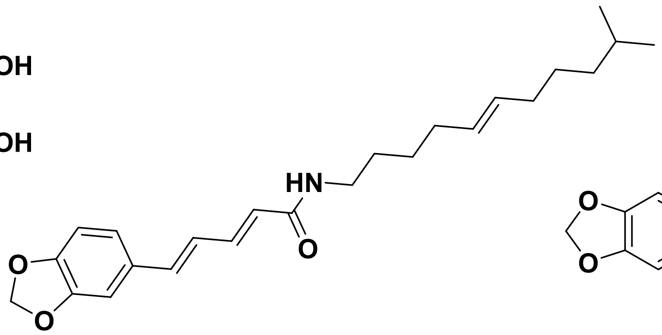

(**201**)

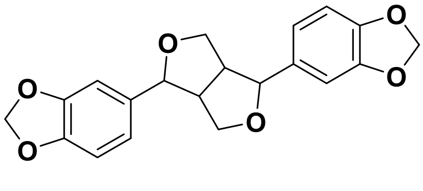

(**202**)

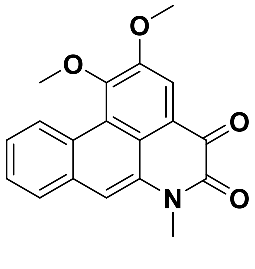

(**203**)

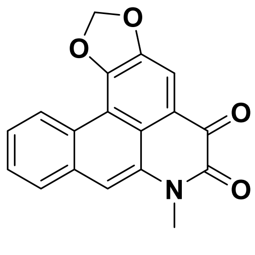

(**204**)

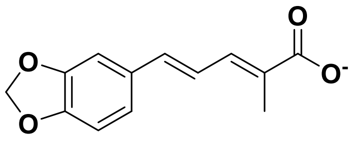

(**205**)

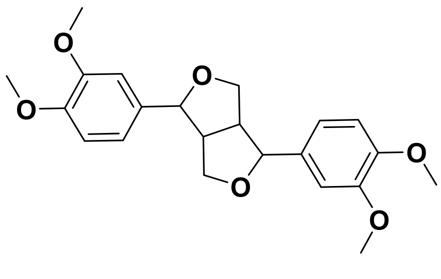

(**206**)

Figure S9. *Cont.*

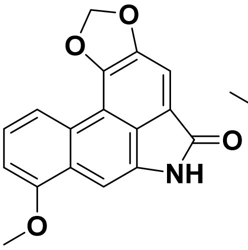
(**207**)

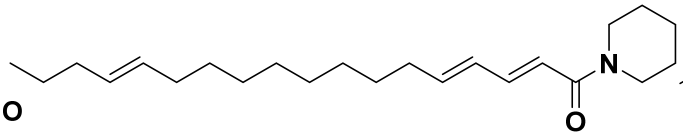
(**208**)

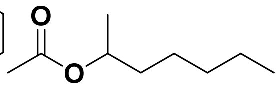
(**209**)

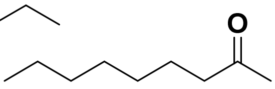
(**210**)

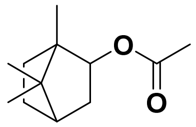
(**211**)

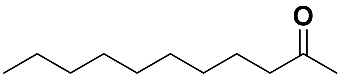
(**212**)

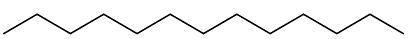
(**213**)

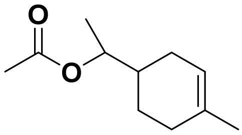
(**214**)

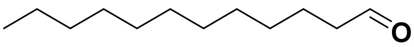
(**215**)

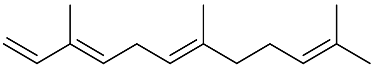
(**216**)

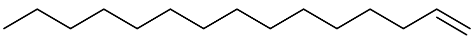
(**217**)

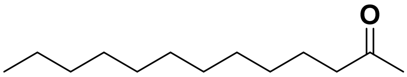
(**218**)

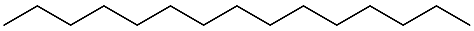
(**219**)

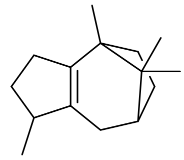
(**220**)

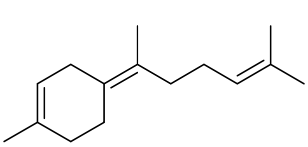
(**221**)

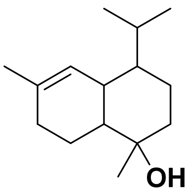
(**222**)

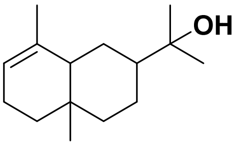
(**223**)

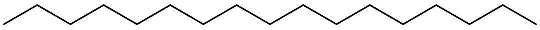
(**224**)

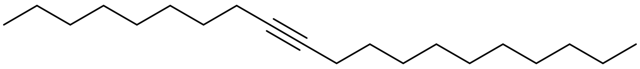
(**225**)

Figure S9. The structure chemical compound of *P. longum*

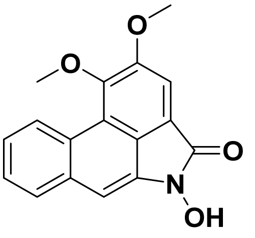

(**226**)

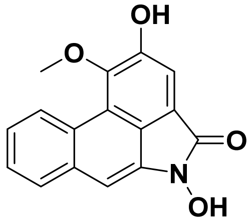

(**227**)

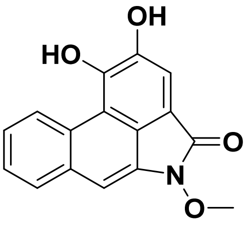

(**228**)

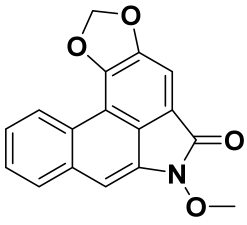

(**229**)

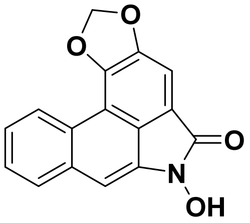

(**230**)

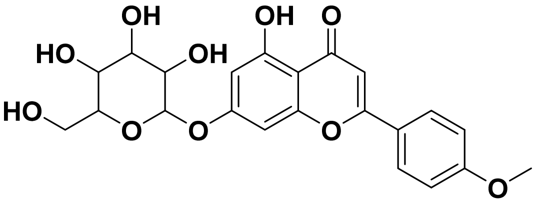

(**231**)

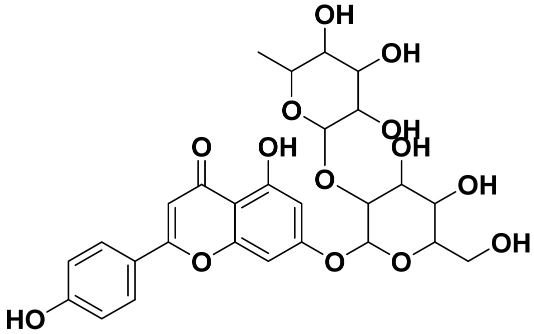

(**232**)

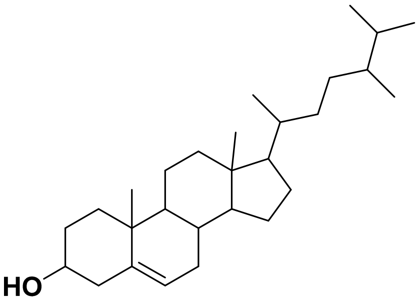

(**233**)

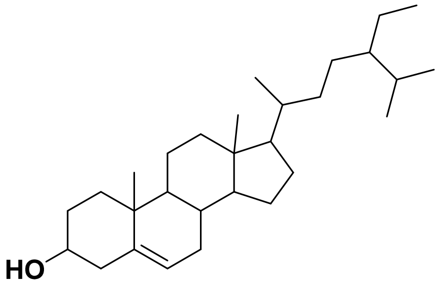

(**234**)

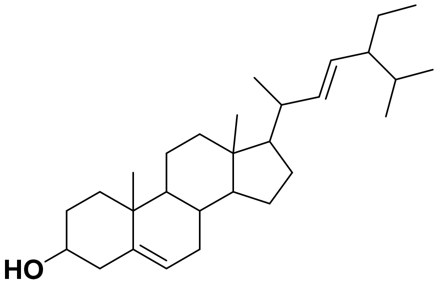

(**235**)

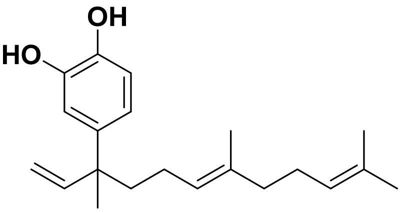

(**236**)

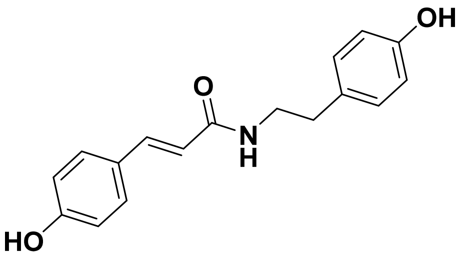

(**237**)

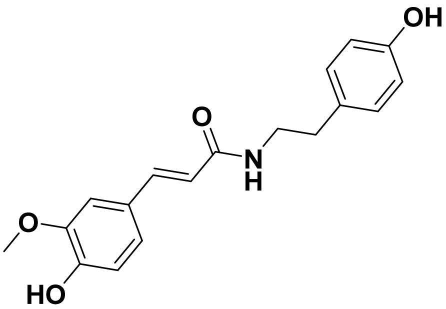

(**238**)

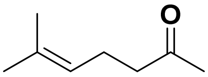

(**239**)

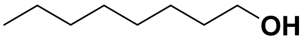

(**240**)

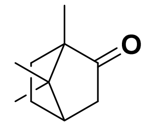

(**241**)

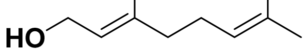

(**242**)

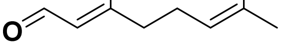

(**243**)

Figure S10. *Cont.*

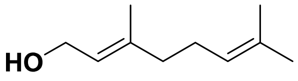

(**244**)

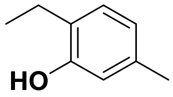

(**245**)

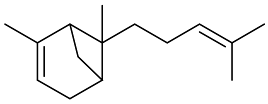

(**246**)

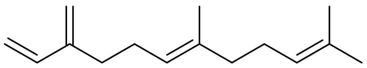

(**247**)

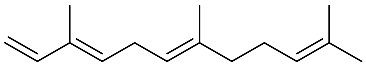

(**248**)

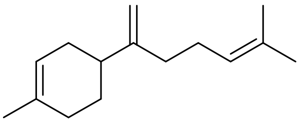

(**249**)

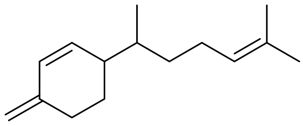

(**250**)

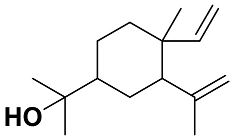

(**251**)

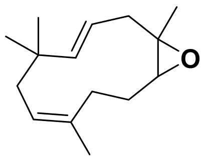

(**252**)

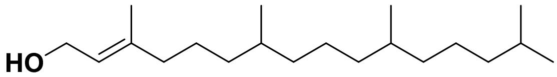

(**253**)

Figure S10. The structure chemical compound of *P. umbellatum*

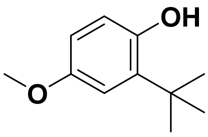

(**BHA**)

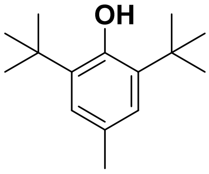

(**BHT**)

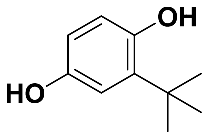

(**TBHQ**)

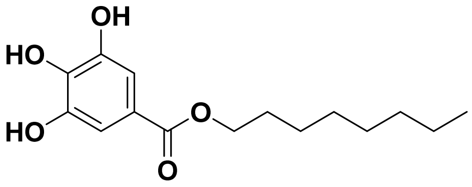

(**OG**)

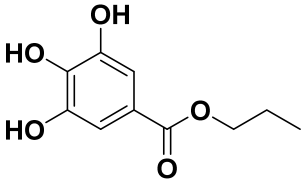

(**PG**)

Figure S11. The chemical structure of synthetic antioxidants
